# A guide to preprinting for early-career researchers

**DOI:** 10.1242/bio.059310

**Published:** 2022-07-25

**Authors:** Cassandra L. Ettinger, Madhumala K. Sadanandappa, Kıvanç Görgülü, Karen L. Coghlan, Kenneth K. Hallenbeck, Iratxe Puebla

**Affiliations:** 1 Department of Microbiology and Plant Pathology, University of California, Riverside, CA 92521, USA; 2 Department of Molecular and Systems Biology, Geisel School of Medicine at Dartmouth, Hanover, NH 03755, USA; 3 Comprehensive Cancer Center Munich, Klinikum rechts der Isar, Technical University of Munich, 81675, Munich, Germany; 4 George C. Gordon Library, Worcester Polytechnic Institute, Worcester, MA 01609, USA; 5 TerraPrime, Danvers, MA 01923, USA; 6 ASAPbio, Cambridge, UK

**Keywords:** Preprints, Early-career researchers, How-to guide, Open science, Advice, Life sciences

## Abstract

The use of preprints, research manuscripts shared publicly before completing the traditional peer-review process, is becoming a more common practice among life science researchers. Early-career researchers (ECRs) benefit from posting preprints as they are shareable, citable, and prove productivity. However, preprinting a manuscript involves a discussion among all co-authors, and ECRs are often not the decision-makers. Therefore, ECRs may find themselves in situations where they are interested in depositing a preprint but are unsure how to approach their co-authors or advisor about preprinting. Leveraging our own experiences as ECRs, and feedback from the research community, we have constructed a guide for ECRs who are considering preprinting to enable them to take ownership over the process and to raise awareness about preprinting options. We hope that this guide helps ECRs to initiate conversations about preprinting with co-authors and encourage them to preprint their future research.

## Abstract (precis)

**Summary:** Are you an early-career researcher considering preprinting, but unsure how to approach conversations about the possibility? Here, we discuss preprinting and provide tips to enable you to take ownership over the process.

## Introduction

Preprints have attracted the attention of life scientists due to their growth in recent years and their role in facilitating the prompt sharing of research findings related to the COVID-19 pandemic (Fraser et al., 2021). Preprints support the rapid dissemination of research, accelerate scientific progress, and directly benefit individual researchers, particularly early-career researchers (ECRs) including undergraduate students, graduate students, postdocs, research associates, research scientists, junior group leaders, staff scientists, and other researchers. In addition to offering more control over how and when to share research work compared to publication at a journal, preprints enable researchers to present their research contributions to funding agencies and hiring committees while the manuscript is undergoing the editorial process at a journal.

Though ECRs are often interested in open science and preprints (Sarabipour et al., 2019; Wolf et al., 2021), many find themselves in situations where the decision on how to publish their research does not lie solely with them. Whether to preprint a manuscript involves a discussion among co-authors, and the ECR's advisor, the group leader, or the corresponding author will often make the final decision. Therefore, ECRs may find themselves in a situation where they would like to preprint but are unsure how to approach their advisor about preprinting. Drawing on our own experiences as ECRs and feedback from the research community, we have constructed the following guide for ECRs interested in preprinting their research. In this guide, we focus on: (1) what preprints are and current trends in the life sciences, (2) how to approach conversations about preprints with co-authors and advisors, (3) common concerns about preprinting, (4) practical steps for depositing preprints, and (5) how to get involved with preprints more broadly. Besides raising awareness, we hope that the resources and suggestions in this article will be informative and helpful to ECRs in understanding the advantages of preprints.

## Do your research: what is a preprint?

A preprint is defined as a full draft version of a research manuscript shared publicly prior to the peer-review process (Tennant et al., 2018 preprint; Mudrack, 2020). Posting a preprint serves as a public, permanent disclosure of one's research. In patent terms it would serve as prior art, assigning a date in the scholarly record for any subsequent discussion of who found a particular result first. Preprints are assigned a persistent identifier, most commonly a digital object identifier number (DOI), which allows them to become a permanent part of the scholarly record (International DOI Foundation, 2021). The DOI records metadata for ease of discoverability. Many funders, such as the National Institute of Health (NIH) in the US, the European Research Council, or the Australian Research Council, now allow preprint citations in grant applications or reports (Kaiser, 2017; Watson, 2021). The preprint can be cited in subsequent papers furthering the scholarly record and making research results available in a timely manner.

Preprints can enhance the reachability and visibility of research findings, as they are not associated with access barriers (Fraser et al., 2020). Thus, preprints enable open science as the servers are free-to-use and free-to-access, thereby facilitating early discovery and global public engagement (Maggio et al., 2018; UNESCO Recommendation on Open Science, 2021). Preprints also support an international and equitable scientific community: there is no paywall, which means that researchers can read and cite work they otherwise would not be able to access due to barriers caused by journal subscription fees.

Preprints are not new to the research community. In the 1960s, the NIH created the Information Exchange Groups (IEGs) to circulate copies of biological preprints. The IEGs ended up growing into seven different groups with a membership of more than 3600 participants and distributed over 2500 documents. However, by 1967 the IEGs were abandoned after several journal publishers refused to accept articles circulated as preprints (Cobb, 2017). Physicists experimented with similar models, and in 1991, arXiv was founded as a repository for manuscripts in the physical sciences (ArXiv, 2021). While physicists adopted preprints to disseminate work with colleagues, preprints in the life sciences did not take off until the 2010s, with the start of bioRxiv and initial signs of support by funders and publishers (Puebla et al., 2022).

### Preprint servers and landscape

Preprint adoption in the life sciences started with the launch of bioRxiv in November 2013. Currently, over 50 preprint servers cover a wide range of disciplines; for a list of preprint servers relevant to life sciences, biomedical, and clinical research, refer to the ASAPbio webpage (https://asapbio.org/preprint-servers; Kirkham et al., 2020). While these servers follow different governance models, they are operated by academic communities, academic institutions, or publishers. Similar to journal publications, searching for preprints is straightforward, as Google Scholar and Europe PMC index many preprint servers including bioRxiv, Research Square, and medRxiv. This means that many of the ways that one uses to keep up with published literature (for tips see Pain, 2016) can also alert you to the latest preprints.

**Fig. 1.**
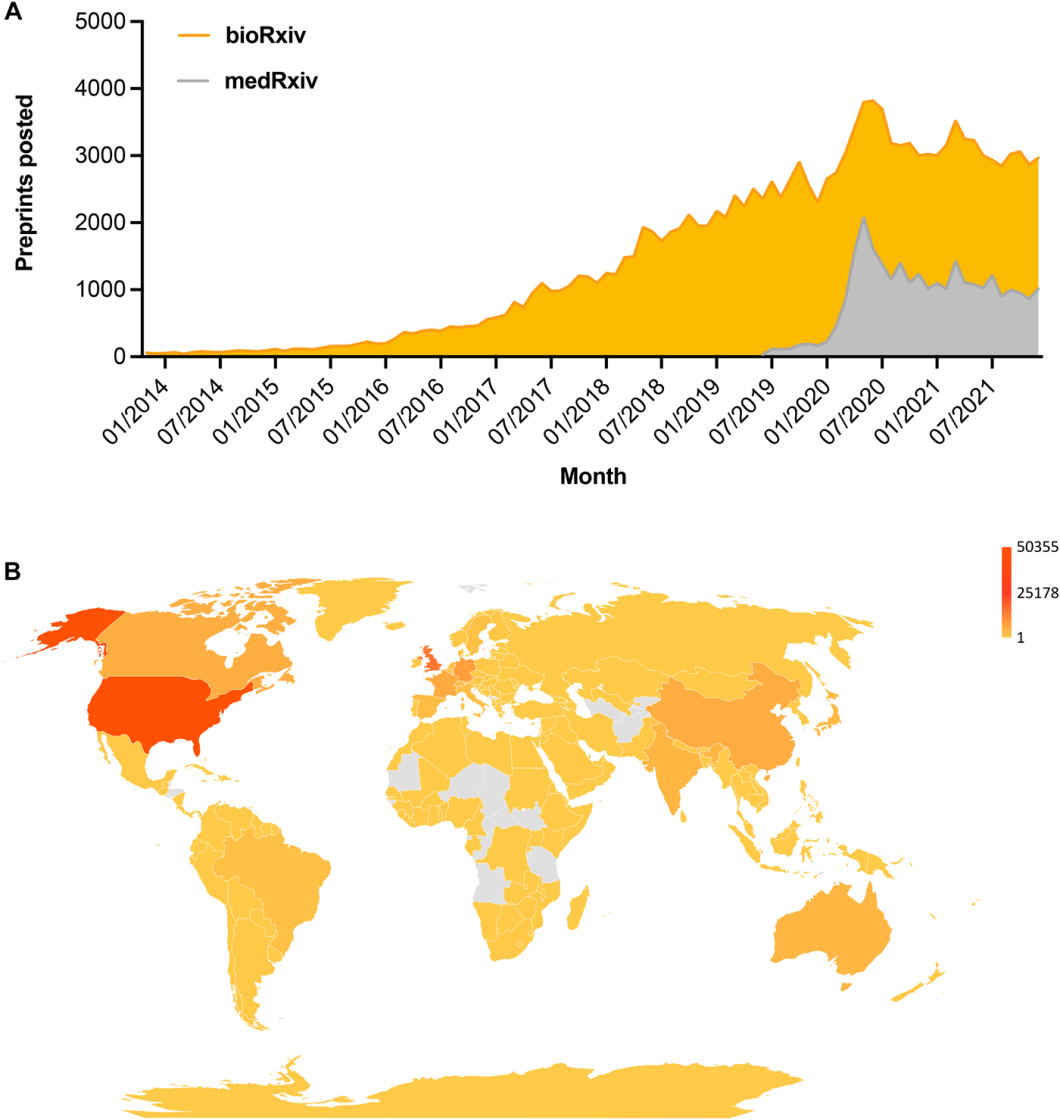
(A) Monthly new submissions to bioRxiv (orange - November 2013 to December 2021) and medRxiv (grey - June 2019 to December 2021). (B) A heat map showing the country-wise distribution of preprints in both bioRxiv and medRxiv based on the institutional affiliation of the corresponding author. The color coding uses a log scale. (Data curated from bioRxiv and medRxiv- from servers launch untill August 2021).

The number of cumulative submissions to preprint servers over time demonstrates increased acceptance of preprinting among life science researchers (Tennant et al., 2018 preprint); for the evolution of life science preprints in that time period, see the data indexed by Europe PMC (Europe PMC, 2021). bioRxiv, the largest biology preprint server, had cumulatively published over 200,000 preprints by early 2022 (Fig. 1A; bioRxiv reporting, 2021). Their sister server medRxiv launched in June 2019 for health sciences, now hosts over 40,000 preprints (Fig. 1A). Researchers from over 170 countries have deposited preprints in bioRxiv, with the majority of preprints originating from the USA and the UK (Fig. 1B) (Abdill et al., 2020). Previous studies looking at the country distribution of preprints before and during the COVID-19 pandemic, also highlight that the US, China and countries in Western Europe are the most represented in bioRxiv and medRxiv (Abdill et al., 2020; Fraser et al., 2021). Disparities in preprint deposition across countries relative to their overall scientific output suggest that geographical barriers may exist to preprint adoption (Abdill et al., 2020).

Consideration of preprint servers based on discipline, scope, policies, and readership is relevant to inform where to deposit your preprint, and in turn to maximize visibility for the work and opportunities for feedback from researchers in your specific field. Data suggests that the adoption of preprints varies from one discipline to another within the life sciences. Neuroscience, microbiology, bioinformatics, cell biology and evolutionary biology are among the fields most extensively represented in bioRxiv (Abdill and Blekhman, 2019; bioRxiv reporting, 2021), whereas infectious diseases, epidemiology, and public and global health preprints are strongly represented in medRxiv (bioRxiv reporting, 2021). The strongest disciplines in medRxiv closely overlap with those relevant to COVID-19 research, as many researchers shared their preliminary data related to COVID-19 in the form of preprints to help inform the response to the pandemic. During the initial months of the pandemic there was not only a surge in the deposition of preprints but also in public engagement with preprinted COVID-19-related research. COVID-19 preprints also received more citations, reactions on social media and coverage in the press compared to non-COVID-19 preprints (Fraser et al., 2021).

Engagement with preprints can also vary according to the server and whether it is predominantly linked to a journal's submission process (Kirkham et al., 2020). Researchers seeking to share their work with their communities before or in parallel to journal submission may post to community-operated servers such as bioRxiv, medRxiv or servers that serve regional communities such as AfricArxiv, RINarxiv or IndiaRxiv. On the other hand, some researchers post their preprint upon journal submission, by opting into services offered by journals to post at a preprint server their publisher runs or has a partnership with. Examples of this type of service include Cell Sneak Peak and Preprints with the Lancet (owned by Elsevier) offered by journals in the Cell and Lancet families, or journals in the Springer Nature portfolio, which offer authors the option to deposit at Research Square, a server partnered with the publisher.

## I am thinking about preprinting my paper - how should I approach it with my advisors and co-authors?

### Talking to your advisor, colleagues, and co-authors

**Fig. 2.**
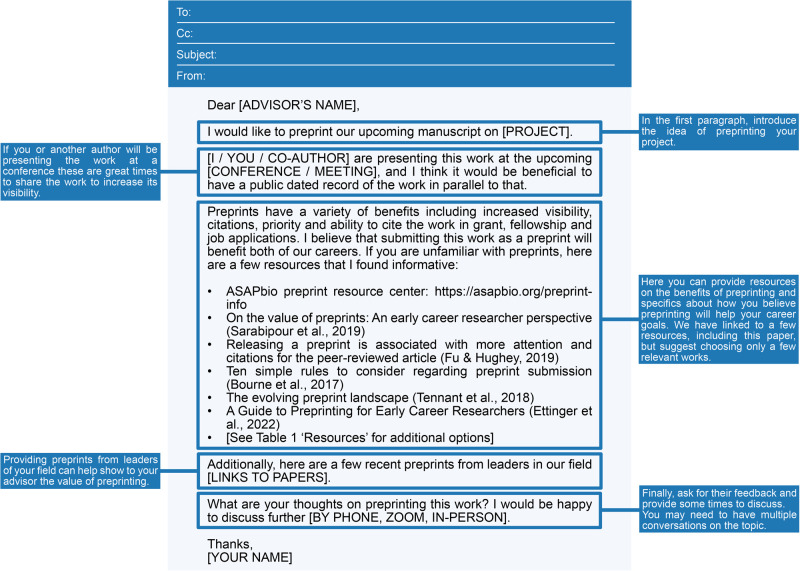
**Draft email to one**’**s advisor.** An email template to help with initiating conversations about preprinting with one's advisor. We have included the same template and a template for emailing co-authors in text format in the supplementary materials (Text S1).

So, after considering all the above, you would like to preprint your paper; how to get started? As a first step, have a conversation with your advisor about preprinting your next paper. If you are unsure about where they stand regarding preprints, you can start by asking about their views on preprinting. If you have these discussions with your advisor or co-authors by email, we have provided some draft email structures to help you (Fig. 2; Text S1). Here are a few important things to consider:

- Keep it simple.
- Familiarize yourself with your institution or funder policy for communicating the work. Do they encourage or require preprints?
- Find out your advisor's priorities for sharing the group's work.
- Provide examples of other researchers in your field who have preprinted.
- Offer additional resources or seek further input about using preprints.

If you are meeting with your advisor in person, even if you come prepared with all the answers, remember that your advisor may have questions that you did not anticipate or may still be unsure of what might be best for the work after your conversation. They may need time to mull over the options and get back to you; not everything needs to be settled in one conversation. You could offer to gather more information on preprinting or their specific concerns to share with them and then continue the conversation at the next meeting. All authors must be on board to preprint the manuscript, so having these meetings early on can leave time for you to address concerns.

In addition, consider the language and construction of the argument that you will use in your preprinting conversations. Try to use ‘I’ language when discussing your goals and motivations and remind all parties how this aligns with your values or will benefit your career. If someone has a different opinion on preprinting than you do, investigate this opinion further by asking them how they reached that conclusion. Come prepared with resources to share and be aware of common concerns (see below and Table 1), but do not pressure your advisor or colleagues to decide right away. Be ready to compromise and table the discussion to be followed up with in the future.

**Table 1.**
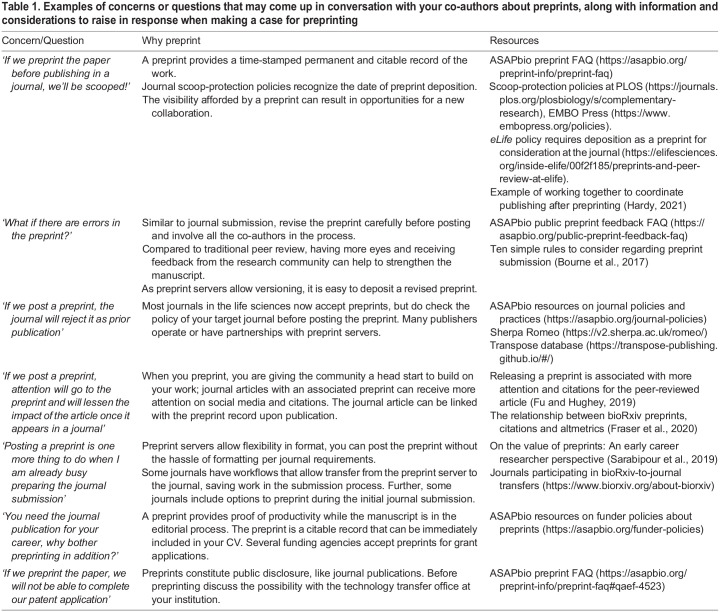
Examples of concerns or questions that may come up in conversation with your co-authors about preprints, along with information and considerations to raise in response when making a case for preprinting

### Construct your argument - what concerns may come up in conversations about preprints?

Several concerns or issues may come up in conversations with co-authors, colleagues, advisors, or others in the community. These issues might be influenced by research field, career stage, or experience. For example, those working in medical fields may raise concerns about sharing findings that may affect patients before peer-review; the stakes in patient treatment and public health are higher than in other fields. Preprint opinion may also differ depending on the level of acceptance of preprints in a discipline. For instance, in research fields with strong preprint adoption, it is less likely to receive the response ‘I did not see your work!’ when you preprint. On the other hand, concerns about visibility or scooping may be more significant for fields with relatively lower adoption or acceptance of preprints.

We outline below (Table 1) some of the concerns or questions that may arise during discussions about preprints. In addition, we explore two of the most common themes in greater detail: scooping and sharing the work before the journal peer-review process.

## Concern #1: I'll get scooped

A common concern among researchers is the risk of scooping – that another competing group will see the preprint and rush to publish their results in a journal before the preprint authors can do so themselves, thereby depriving the preprint authors of the career benefits of publishing in their target journal (Bourne et al., 2017). Interestingly, there is no evidence that the prevalence of scooping in preprints is higher than in the context of journal publications. For instance, in the 2019 bioRxiv survey, only 0.7% of respondents indicated that preprinting prevented them from publishing in their journal of choice (Sever et al., 2019 preprint).

Most remarkably, researchers have used their preprints as an opportunity to initiate collaborations with other groups in the field or to coordinate the publication of their work together, thereby avoiding concerns about priority claims. For example, Dr Josh Hardy discussed how upon seeing a preprint from another group, they got in touch with the preprint authors. The two groups coordinated the journal publication of their respective papers, which ended up appearing in the same journal (Hardy, 2021).

Preprinting allows researchers much more control of when they disseminate their work and is thus an opportunity to prevent being scooped while waiting for the paper to be published in a journal. In addition, preprints provide an avenue for researchers in rapidly moving fields to promptly share their work with their community, where the delay associated with peer review may come at the cost of priority. In the bioRxiv survey, 28% of respondents stated that preprints helped them stake a priority claim in their field (Sever et al., 2019, preprint).

### Preprints enhance visibility

Visibility is an important element in the context of scooping concerns: preprints must be readily discoverable by researchers in the field, which in turn, allows attributing credit to the authors. Will the preprint be seen by colleagues in the field? Or is there a risk that the preprint may be overlooked, and competitors may not cite it?

In the bioRxiv survey, 74% of respondents stated that preprinting increased awareness of their research (Sever et al., 2019, preprint). Preprints are readily searchable online, as indexing services and literature search tools increasingly incorporate them (Scopus, Google Scholar, Europe PMC, and Crossref all index preprints). In addition, authors can quickly disseminate preprints on social media platforms. For example, Twitter plays an important role in increasing the visibility of preprints, with many research groups sharing their latest preprints via Twitter or commenting on colleagues’ latest preprinted work (Chiarelli et al., 2019). Furthermore, social media platforms can allow scientists to immediately measure the community's reactions and engagement with the work by the number of tweets, re-tweets, and likes the preprint receives. Many authors now post Twitter threads highlighting the main findings of their preprints or journal articles. In fact, before writing this guide we used a Twitter thread with polls to gauge ECR interest in preprinting, with 92.5% of respondents recommending preprinting to ECRs (*n*=40) (Fig. S1, Table S1). If you are new to social media, there are several existing guides for scientists that can help you get started (Bik and Goldstein, 2013; Heemstra, 2020; Cheplygina et al., 2020).

In addition, studies have shown that posting preprints results in more attention on social media and a higher number of citations for the article once it appears in a journal (Fu and Hughey, 2019). Altmetric scores are generally higher for articles deposited as preprints; journal publications that have associated bioRxiv preprints receive more mentions on blogs and Wikipedia than non-deposited articles, as well as more mentions in Twitter or Mendeley (Abdill and Blekhman, 2019; Fraser et al., 2020). COVID-19 preprints have also been widely reported in the lay media (Fleerackers et al., 2022). The early accrual of citations for the journal publication suggests that the community had already taken note of the preprint, which gave them a chance to consider the work as part of their own research between the preprint appearance and the journal publication.

### Preprints establish priority

An important step in the research process is to disseminate your findings to the scientific community, and in turn, be able to claim credit for the work. Recognition for research productivity is essential to establishing a reputation in the field, acquiring grants, and career progress. A preprint provides a permanent time-stamped record for the research findings in a much shorter timeline than a journal publication. Thus, when time is critical (e.g. when completing your thesis or finishing a project before moving to another position), preprinting can greatly benefit ECRs.

In the coming years, life scientists might use preprints as a channel to establish priority, which has been established practice in the physics community for years (Vale and Hyman, 2016). In support of this idea, several publishers such as EMBO Press, PLOS, and eLife have *‘*scoop protection’ policies that recognize the date of the preprint deposition as the date at which their policy applies. The scooping-protection policy stipulates that from the date of the preprint, if another publication appears reporting similar findings, that would not impact the consideration of the paper submitted to their journals.

Researchers often worry about the potential risk of scooping when they present their preliminary findings at conferences or symposiums. Attendees could use the information they heard at the conference and scoop the presenter. As the information would have been available only to the conference attendees, there is limited audience to vouch for who has priority over that work and it would not be easy to establish who did what and when. Depositing a preprint before the conference presentation records the priority claim with a time-stamp and provides protection from scooping.

### Preprints are citable

A tangible benefit of preprints is that they are citable and can prove productivity for prospective funders. Many funding agencies now have policies that allow citing preprints as part of grant applications and reports (more information on funder policies at asapbio.org/funder-policies). We expect to see more funding agencies update their policies, recognizing the importance of preprints in the future. Besides funders, several research institutions have started to include preprints in their processes for hiring and promotion (see asapbio.org/university-policies).

## Concern #2: My work hasn't been peer reviewed yet

Another common concern that may arise in conversations around preprints is sharing work before peer review. Some researchers worry about disseminating their findings before completing the traditional peer-review process, which provides feedback on the work and can also address any errors before the broader circulation of the manuscript. It is important to note that the preprint should be carefully prepared before depositing it to the server, similar to journal manuscript preparation. To this end, ensure that all co-authors check the paper before posting and consider receiving feedback from colleagues prior to submitting the paper to the preprint server.

### Preprint feedback focuses on the science and not on journal fit

An advantage of posting a preprint is that feedback received from the scientific community can help to improve the manuscript and is independent of subjective evaluations about journal fit. Incorporating community feedback into the manuscript can even increase the chances of eventual publication. A preprint brings more eyes and a broader range of perspectives to the paper than the traditional two or three reviewers from the journal's peer-review. Thus, it can provide a robust mechanism to identify any issues before a manuscript enters the journal's editorial process and valuable input on specific aspects including the statistical analyses, methodology, or the interpretations of the data. Importantly, preprint servers allow authors to submit new versions of the preprint. It is straightforward for authors to post a revision as a new preprint version after incorporating additional work or correcting any oversights. The mechanisms for preprint versioning allow updates or corrections to the paper in a faster and simpler path compared to corrections to the article's version of record at a journal.

### Preprints enable journal-independent peer-review

Several platforms offer feedback and evaluations on preprints, and in some of these the peer-review process runs similarly to the traditional journal peer review. For example, Review Commons, an initiative by EMBO Press and ASAPbio, allows researchers to submit their preprint for peer review prior to journal submission. Review Commons has partnered with 17 affiliate journals — the Company of Biologists’s journals, EMBO Press journals, PLOS, *eLife*, *Journal of Cell Biology*, and *Molecular Biology of the Cell* — that have agreed to use the reviews provided by Review Commons to inform their evaluation and editorial decision, thus avoiding multiple review rounds. Review Commons requires the authors to post a preprint before submitting the manuscript to an affiliate journal.

Services such as Review Commons and Peer Community In - which also completes evaluation of preprints - involve the review of preprints in a process coordinated by an editor or similar role. On the other hand, other platforms, such as PREreview and PubPeer, allow any community member to provide feedback on the preprint (Table 2). In addition, many preprint servers offer commenting features that allow readers to contribute comments on preprints in a variety of formats; such comments may involve praise for the work, queries to the authors, comments on specific aspects of the study, summaries from journal club discussions or even copies of full reviews for the preprint (Malički et al., 2021).

**Table 2.**
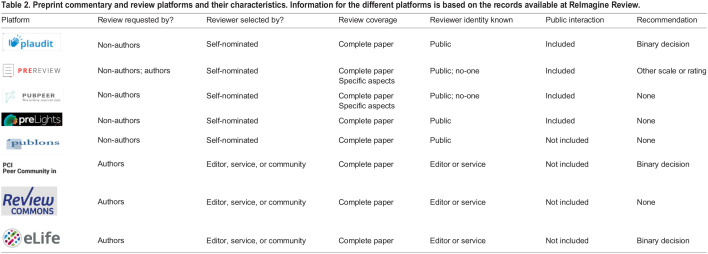
Preprint commentary and review platforms and their characteristics. Information for the different platforms is based on the records available at ReImagine Review.

Public comments posted on the preprint can also help inform and positively shape the editor's decision upon manuscript submission to a journal. Some journals such as *Proceedings of the Royal Society B* and *Open Biology* have appointed preprint editors who check the latest preprints to solicit submissions to their journals (Neiman et al., 2021).

### Preprints generally change little upon journal publication

A majority of the manuscripts posted as preprints go on to be published in a journal; a study of bioRxiv preprints found that two thirds of the preprints appeared at a journal within 2 years (Abdill and Blekhman*,* 2019). Additional studies that have evaluated the content of preprints and their associated journal publications found that the reporting quality in preprints is within a similar range as that of peer-reviewed articles (Carneiro et al., 2020) and that the main content and conclusions changed little between the preprint and the journal publication for the same work (Brierley et al., 2022; Nicholson et al., 2022; Zeraatkar et al., 2022). These studies suggest that there is no evidence to consider research findings reported via preprints as less trustworthy than journal publications. The peer-review process at journals provides a valuable mechanism to scrutinize research work and identify potential flaws or oversights, but it is important to remember that peer review is not infallible (Schroter et al., 2008), and the ‘peer reviewed’ label does not imply that a particular published finding is reliable; all research works should be critically appraised, whether they appear at a journal, at a preprint server or in another format.

## Next steps - how to preprint your paper?

**Fig. 3.**
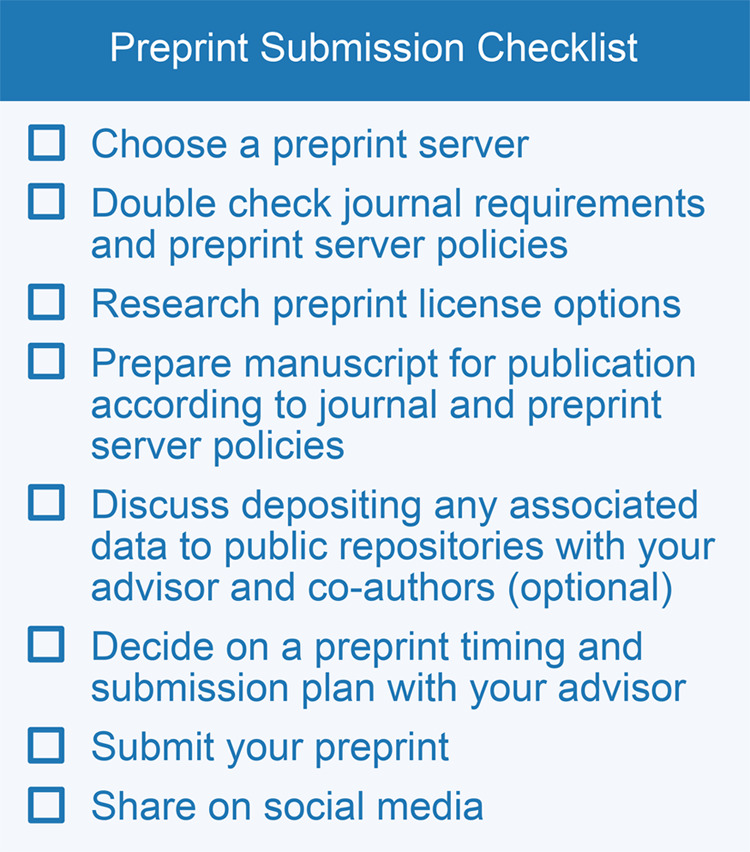
**Preprint submission checklist.** A suggested checklist to help with preprint submission after having a successful conversation and the green light from advisors and co-authors to preprint.

Once you have your co-authors’ green light to preprint the work, here are a few actionable steps to complete the preprint deposition (Fig. 3).

### Preprint server

First, you need to choose a preprint server for your manuscript. Think carefully about your audience and what server will best reach the targeted audience (see above). If you plan to submit the manuscript to a journal, familiarize yourself with the journal's editorial policies about preprints. Check if the journal specifies any preprint servers they accept for preprint deposition, for example, some journals have policies only allowing preprints to be deposited on non-profit servers (e.g. *bioRxiv, AfricaArXiv*).

### Preprint license

It is also important to think about the license you will apply to the preprint. You have several options - from retaining all rights (i.e. meaning you do not give default permission to reuse the work) to a range of Creative Commons (CC) licenses, which standardize permissions for the type of use allowed for the work (asapbio.org/licensing-faq). A CC BY license allows any type of re-use without requiring permission from the author, providing credit is given to the original author(s). This type of credit is called attribution (AboutCCLicenses, n.d.). The CC BY license is the most common type and its designation has been shown to increase citation and visibility of monographs (Snijder, 2015). There are additional license options that can be used to preserve copyright, the more licenses options chosen increases the restrictions on reuse: CC BY-NC (cannot be used for commercial purposes), CC BY-ND (non-derivative, must be shared in its original form) and CC BY-SA (share-alike, if re-used must be published under the same or a more restrictive license). These license options (BY, NC, ND, and SA) can be chosen in combination to retain rights and further specify reuse restrictions (e.g. CC BY-NC-SA, etc). While some preprint servers offer a range of license options (e.g. bioRxiv, medRxiv, OSF Preprints), others require a CC-BY license (e.g. Research Square, preprints.org, SciELO Preprints).

### Preprint preparation

In general, preprint servers are format agnostic, meaning they accept a single file of your manuscript in any format (for example, a single PDF file in the formatting style of the journal of your choice!) and then authorship information. You can link the preprint-related data and additional resources deposited in public repositories to your preprint. This may be important if your target journal has an open-data policy (e.g. ASM journals, BMC-series journals) which requires all data and code to be publicly available.

### Preprint submission

Now that you've chosen a preprint server, license type, and prepared your manuscript, decide who will submit the manuscript and when it will be submitted. In the bioRxiv survey, authors preferred preprinting either before journal submission (42%) or concurrent to journal submission (37%) (Sever et al., 2019 preprint). Some journals work with preprint servers, like bioRxiv, to also allow for direct submission of your manuscript to a journal after posting to the preprint server. After the preprint submission, don't forget to share your new preprint on social media (Heemstra, 2020; Cheplygina et al., 2020)!

### If your co-authors aren't interested in preprinting this time...

Irrespective of the field, many researchers are still wary of preprinting, and it is understandable that other authors may have concerns or may need additional time to consider your request. Almost half of the respondents in our Twitter survey who were unable to convince their co-authors to preprint, indicated that their co-authors might be open to preprinting in the future. Offer to continue the conversation another time and suggest to them that it's worth keeping an eye on the latest preprints coming out in your field. You may also suggest you revisit the option of preprinting for another paper where they may view the stakes as less high. If your co-authors are still uninterested, there are still many other ways to get involved with preprints even if you are unable to preprint your current work.

## Other ways to get involved with preprints

Beyond providing an opportunity to promptly share your work and get credit for it, preprints also offer other benefits to your scientific career. For example, several communities with an interest in open science also support preprints. Getting involved with one or more of those groups can be a way to expand your professional network and connect with other researchers in your discipline.

ASAPbio has an international community of researchers and others in the science communication space, who drive initiatives to support preprints and interact and support each other. ASAPbio also runs a fellows program allowing participants to learn more about preprints and develop skills to drive discussions about the productive use of preprints in the life sciences. eLife coordinates an ambassadors program, which aims to bring together ECRs interested in promoting change in academic culture and science communication. preLights, an initiative of the Company of Biologists, provides a platform for ECRs to highlight preprints they find of interest and is another way to engage with preprints.

If you are interested in developing your review skills, several options are currently available. Preprint journal clubs are an excellent opportunity to keep up to date with the latest research in your field and connect with others. If you are part of a local journal club, you can suggest incorporating preprints, if they are not already covered. If you do not have a local journal club, you can explore online options, e.g. PREreview coordinates live-streamed preprint journal clubs.

## Conclusion

We hope that this informational guide will be useful for readers, especially ECRs, interested in preprinting their research. In addition to exploring the current landscape of preprints in the life sciences, we have discussed common concerns around preprints that might come up in conversations with colleagues. The tips provided in this article are useful for having conversations with advisors and co-authors about preprinting, including email templates and practical steps needed to preprint your work.

In this piece, we may have missed many tips and suggestions, but as preprints continue to grow, so will our collective expertise as well as the evidence around the use of preprints for science communication. We are excited to watch the preprinting community continue to grow and look forward to seeing more preprint engagement from ECRs in the coming years.

## Supplementary Material

Supplementary information
