# Supplementary material for: A guide to preprinting for early-career researchers
Source: Biol Open. 2022 Jul 25;11(7):bio059310. doi: 10.1242/bio.059310 (PMC9346271; doi:10.1242/bio.059310)
Supplement: Supplementary information [file biolopen-11-059310-s1.pdf]

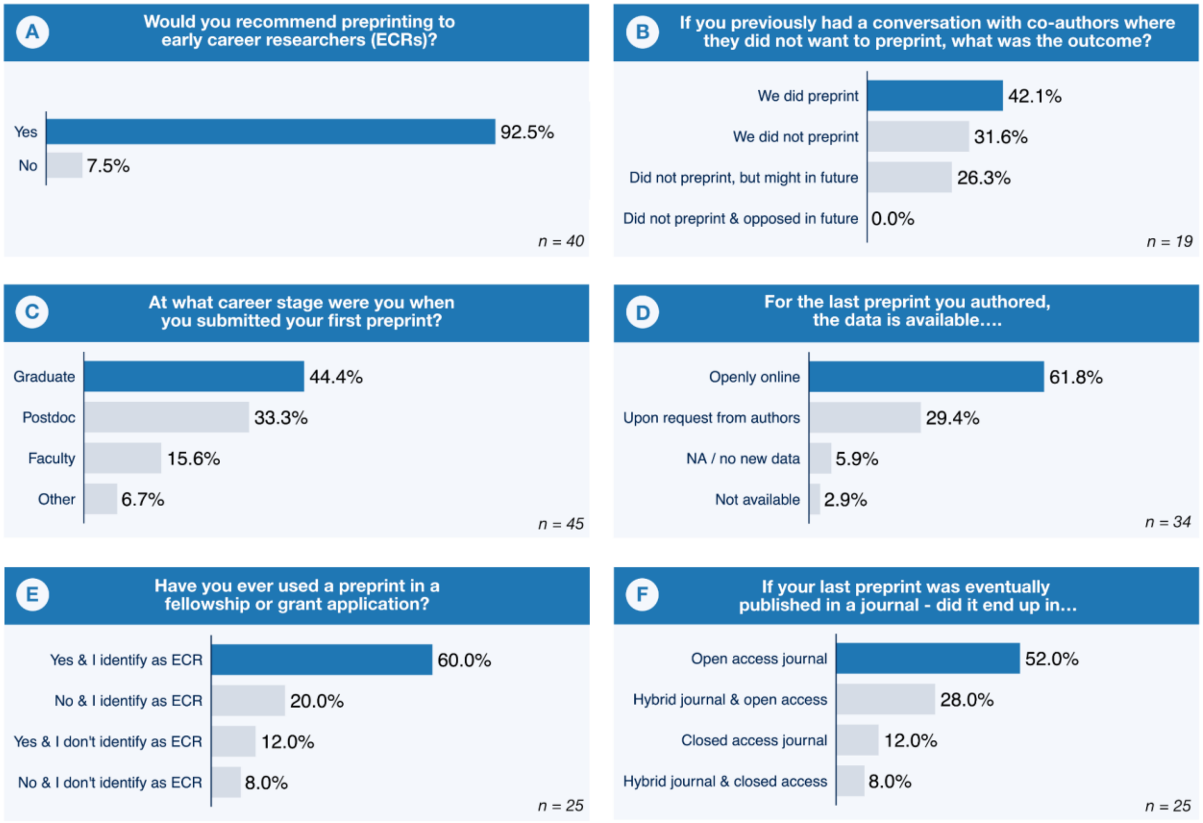

Fig. S1. Twitter thread poll results. On August 30, 2021, we posted six multiple-choice survey questions (**A-F**) to Twitter to understand perceptions about preprints by some Twitter users and their experiences with preprinting (Table S1). Here we show the resulting percentage of responses to each question. The total number of respondents per question is notated in the bottom right corner of each panel, ranging from 19 to 45 responses per question. While limited and not generalizable, the results of these polls illustrate how Twitter can be used by ECRs to engage with a scientific audience.

Biology Open • Supplementary information

Table S1. Twitter thread example. On August 30, 2021, we posted the Tweets included here in numerical order as a single Twitter thread. Tweets #2, 3, 4, 6, 7, and 8 utilized Twitter's internal polling platform and were run for the maximum allowed time of 7 days.

Tweet	Questions
**#1**	Ever posted or considered posting a #preprint? We (Karen Coghlan @madhumalaks @kivancgorgulu @kenkhallenbeck & @casettron) are a group of 2021 @ASAPBio_ fellows interested in #preprinting conversations & we want to hear from you! Please answer the following polls & questions!
**#2**	At what career stage were you when you submitted your first #preprint? Other = Undergraduate, Research Assistant, Technician, Industry position, etc.<br>A. Graduate<br>B. Postdoc<br>C. Faculty<br>D. Other
**#3**	In your experience, would you recommend #preprinting to early career researchers (ECRs)? What are the pros and cons of #preprinting for ECRs? Please comment below or DM.<br>A. Yes<br>B. No
**#4**	If you have previously had a conversation with co-authors where they did not want to #preprint, what was the outcome? Please share their argument below or DM.<br>A. We did preprint<br>B. We did not preprint (DNP)<br>C. DNP but might in future<br>D. DNP & opposed in future
**#5**	What advice would you give to an ECR who is having conversations with their advisor or co-authors about #preprinting? Please comment below or DM.
**#6**	For the last #preprint you authored, the data is available….<br>A. Openly online<br>B. Upon request from authors<br>C. Not available<br>D. NA / no new data
**#7**	If your last #preprint was eventually published in a journal - did it end up in…<br>A. Open access (OA) journal<br>B. Hybrid journal & OA<br>C. Hybrid journal & closed<br>D. A closed access journal

Biology Open • Supplementary information

**#8**	Have you ever used a #preprint in a fellowship or grant application? ECR = undergraduate, graduate student, postdoc or equivalent stages.<br>A. Yes & I identify as ECR<br>B. No & I identify as ECR<br>C. Yes & not ECR<br>D. No & not ECR
**#9**	For the last paper you #preprinted, how did you decide which server to submit to? Please comment below on how you came to your choice or DM.
**#10**	Thank you for sharing your experiences on #preprinting with us. If you would like to share or discuss more, please reach out via DM to any of the awesome members of our 2021 @ASAPBio_ fellows working group @madhumalaks @kivancgorgulu @kenkhallenbeck @casettron

Biology Open • Supplementary information

Text S1: Email drafts (A) initial email to the advisor(s) and (B) after discussing with your advisor(s), a follow-up email draft to send to collaborators.

(A)

Dear [ADVISOR'S NAME],

I would like to preprint our upcoming manuscript on [PROJECT].

[I / YOU / CO-AUTHOR] are presenting this work at the upcoming [CONFERENCE / MEETING], and I think it would be beneficial to have a public-dated record of the work in parallel to that.

Preprints have a variety of benefits including increased visibility, citations, priority, and the ability to cite the work in grants, fellowships, and job applications. I believe that submitting this work as a preprint will benefit both of our careers. If you are unfamiliar with preprints, here are a few resources that I found informative:

- ASAPbio preprint resource center: https://asapbio.org/preprint-info
- On the value of preprints: An early career researcher perspective (Sarabipour et al., 2019)
- Releasing a preprint is associated with more attention and citations for the peer-reviewed article (Fu & Hughey, 2019)
- Ten simple rules to consider regarding preprint submission (Bourne et al., 2017)
- The evolving preprint landscape (Tennant et al., 2018)
- A Guide to Preprinting for Early Career Researchers (Ettinger et al., 2022)
- [See Table 1 'Resources' for additional options]

Additionally, here are a few recent preprints from leaders in our field [LINKS TO PAPERS].

What are your thoughts on preprinting this work? I would be happy to discuss further [BY PHONE, ZOOM, IN-PERSON].

Thanks,
[YOUR NAME]

(B)

Dear [COLLEAGUES / CO-AUTHORS / ALL],

Dr. [ADVISOR'S NAME] and I have discussed and would like to proceed with preprinting our upcoming manuscript on [PROJECT].

[I / YOU / CO-AUTHOR] are presenting this work at the upcoming [CONFERENCE / MEETING], and [I / WE] think it would be beneficial to have a public dated record of the work in parallel to that.

Biology Open • Supplementary information

You may have heard about preprints, as many groups in our field have posted their papers to bioRxiv [or relevant server], see for example these recent preprints that are like our work [LINKS TO PAPERS].

Preprints have a variety of benefits including increased visibility, citations, priority, and the ability to cite the work in grants, fellowships, and job applications. If you are unfamiliar with preprints, here are a few resources that I found informative:

- ASAPbio preprint resource center: https://asapbio.org/preprint-info
- On the value of preprints: An early career researcher perspective (Sarabipour et al., 2019)
- Releasing a preprint is associated with more attention and citations for the peer-reviewed article (Fu & Hughey 2019)
- Ten simple rules to consider regarding preprint submission (Bourne et al., 2017)
- The evolving preprint landscape (Tennant et al., 2018)
- A Guide to Preprinting for Early Career Researchers (Ettinger et al., 2022)
- [See Table 1 ‘Resources’ for additional options]

Please let me know what your thoughts are on preprinting this work and whether we can proceed. I would be happy to share further information or discuss any concerns you have further [BY PHONE, ZOOM, IN-PERSON, GROUP MEETING].

Thanks,
[YOUR NAME]
